# Leafy Vegetable Nitrite and Nitrate Content: Potential Health Effects

**DOI:** 10.3390/foods12081655

**Published:** 2023-04-15

**Authors:** Sanja Luetic, Zlatka Knezovic, Katarina Jurcic, Zrinka Majic, Ksenija Tripkovic, Davorka Sutlovic

**Affiliations:** 1 Teaching Institute for Public Health, Split-Dalmatia County, 21000 Split, Croatia; 2 Department of Health Studies, University of Split, 21000 Split, Croatia; 3 Department of Toxicology and Pharmacogenetics, School of Medicine, University of Split, 21000 Split, Croatia

**Keywords:** nitrate, nitrite, leafy vegetables, legal limits, Swiss chard

## Abstract

The aim of this research was to determine the concentrations of nitrates and nitrites in different types of vegetables that are commonly represented in the diet of the inhabitants of Split and Dalmatian County. Therefore, using the method of random selection, there were 96 samples of different vegetables. The determination of the nitrate and nitrite concentrations was carried out by high-pressure liquid chromatography (HPLC) with a diode array detector (DAD). The nitrate concentrations in the range 2.1–4526.3 mg kg^−1^ were found in 92.7% of the analyzed samples. The highest nitrate values were found in rucola (*Eruca sativa* L.) followed by Swiss chard (*Beta vulgaris* L.). In 36.5% of the leafy vegetables intended for consumption without prior heat treatment, nitrite was found in the range of 3.3–537.9 mg kg^−1^. The high levels of nitrite in the vegetables intended for fresh consumption and the high nitrate values in Swiss chard indicate the need to establish maximum nitrite limits in vegetables, as well as the broadening of legal nitrate limits to wide varieties of vegetables.

## 1. Introduction

Vegetables are a rich source of minerals, vitamins, and carbohydrates, as well as very useful non-nutrients (polyphenols, flavonoids, and glucosinolates) [1,2]. One of the best ways to prevent many diseases, including obesity, which is becoming a significant public health problem, is a Mediterranean diet rich in vegetables. Therefore, the regular consumption of sufficient quantities of vegetables is beneficial for various health outcomes [3,4].

The rising demand for vegetables on the market requires more intensive farming practices and the heavy application of fertilizers, the main source of nitrates. Synthetic mineral fertilizers, such as ammonium nitrate, urea, anhydrous ammonia, and ammonium sulphate, are the most commonly used fertilizers. Due to the convenience of application and high soil solubility, they frequently replace the application of manure in total [5,6,7].

Nitrate is taken up by the plant’s roots and transported to the aboveground organs by the xylem. Several research findings indicate that the leaves contain more nitrates than the roots [8,9]. While nitrate accumulation differs among species, some families are known to be nitrate accumulators, including Brassicaceae (rucola, radish, and mustard), *Chenopodiaceae* (beetroot, Swiss chard, and spinach), *Amaranthaceae* (amaranthus), *Asteraceae* (lettuce), and *Apiaceae* (celery and parsley). The amount of nitrate in vegetables is greatly influenced by environmental factors (atmospheric humidity, water content in the substrate, temperature, radiation, and photoperiod), as well as agricultural factors (nitrogen dosages and chemical forms, availability of other nutrients) [10].

**Figure 1:**
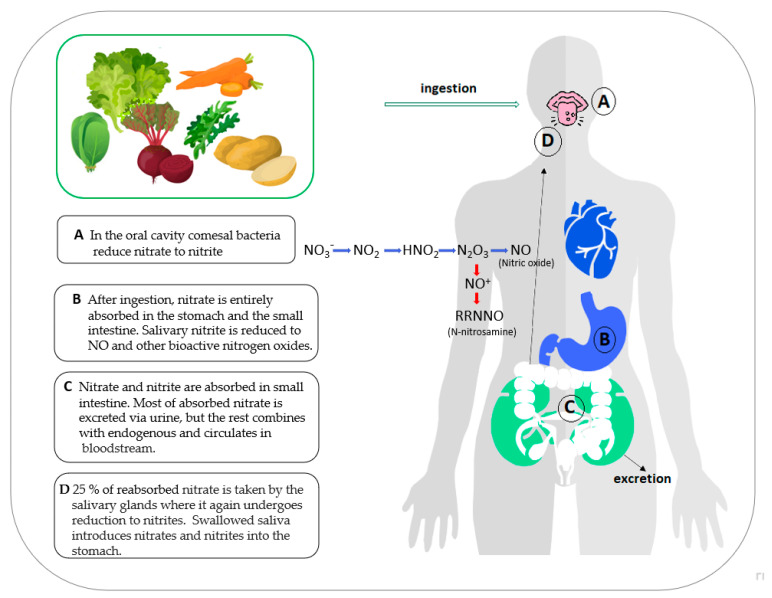
The entero-salivary circulation of nitrate and nitrite (NO_3_^−^, nitrate; NO_2_^−^, nitrite; HNO_2_, nitric acid; N_2_O_3_, dinitrogen trioxide; NO, nitric oxide; NO^+^, nitrosonium ion; RRNNO, reactive nitrogen intermediates).

Although nitrate itself is not poisonous to humans, its metabolites, nitrites, and their reactive nitrogen intermediates can be harmful to our health. Upon intake, nitrate is taken up by the digestive system and transported into the bloodstream, where it combines with endogenous nitrate created by various types of cells throughout the body. The majority of nitrate is eliminated in urine. However, salivary glands absorb approximately 25% of plasma nitrate, leading to high levels of nitrate in the saliva. Salivary nitrate is converted by commensal bacteria to nitrite, which is then further converted to nitric oxide and other reactive nitrogen intermediates, including N-nitrosamine, in the stomach’s acidic environment (Figure 1). N-nitrosamines are carcinogenic and nitrite or nitric oxide can oxidize hemoglobin in red blood cells into an aberrant form methemoglobin. It cannot bind or transport oxygen, so the metabolism of nitrate and nitrite can have detrimental consequences on health [11,12].

Apart from potential adverse impacts, dietary nitrate is a precursor of nitric oxide (NO) which is formed in the acidic environment of the stomach following the NO_3_-NO_2_-NO pathway (Figure 1). NO has an important role in human physiology, particularly in the cardiovascular system and maintenance of neurological functions [13,14].

Endogenous nitric oxide is primarily produced in enzymatic synthesis from semi essential amino acid L-arginine. L-arginine is provided from dietary intakes but also from endogenous pathways through protein turnover and de novo synthesis from citrulline in the urea cycle. In healthy adults with proper nutrition L-arginine can be synthesized in sufficient quantities to meet most normal physiological demands, including NO production [15,16].

Since nitrate is a precursor to NO, numerous studies have looked at the potential positive effects of nitrate-rich foods, particularly vegetables, on the cardiovascular system [17,18]. The results of the studies differ, and there is no clear consensus concerning the possible positive effects of increased nitrate intake [19]. According to the IARC assessment, due to their possible harmful effects, nitrates and nitrites are considered probable carcinogens (2A) [20].

Exposure to nitrates is exogenous, whereas exposure to nitrite is mainly endogenous via nitrate metabolism. According to the European Food Safety Authority (EFSA) survey, the most important sources of nitrate dietary intake are vegetables and fruit contributing 50 to 75% to the overall dietary intake. As for nitrite, the main source is endogenous conversion from nitrate, although in some Central/Southern European countries nitrite levels from vegetables contribute to exogenous nitrite by 11–41%. The conversion factor for the ingested nitrate to nitrite is considered to be 7% [21].

According to the World Health Organization (WHO) and numerous epidemiological studies, a healthy diet with a fruit and vegetables daily intake of 400–500 g is recommended. This can promote good health, lower the risk of different diseases, and strengthen immunity [22,23,24].

The EU Scientific Committee for Food (SCF) and Joint FAO/WHO Expert Committee on Food Additives (JECFA) set acceptable daily intakes (ADI) of 0–3.7 mg kg^−1^ body weight (b.w.) for nitrate ion and 0.07 mg kg^−1^ b.w. for nitrite ion [25,26].

The legislation of the European Union in Regulation 1258/2011 stipulates the maximum permissible concentrations solely for nitrate, whereas the legal regulation is limited just for three types of vegetables (spinach, lettuce, and rucola) [27]. The content of nitrate in other types of vegetables (Swiss chard and cabbage), commonly represented in the nutrition of the Mediterranean population, is not covered by this legislation. Nitrite levels are not prescribed by any regulation.

The aim of our study was to determine the nitrate and nitrite concentrations in randomly selected samples of different vegetables sold at local marketplaces. Mediterranean towns such as Split are characterized by their reliance on local agriculture for their food source. Regarding the use of nitrogen fertilizers and the levels of nitrates and nitrites in their goods, local producers might not adhere to agrotechnical guidelines. Namely, according to data from the Statistical Office of the European Union (Eurostat-European Commission, Directorate-General for Communication) concerning the area under agricultural crops and the consumption of nitrogen fertilizers, Croatia is among the countries with a higher consumption of mineral fertilizers [28]. Mineral fertilizers are used by approximately seventy percent of agricultural households, and this percentage is even greater among commercial businesses. Recently, there have been noticeable shifts, with increasing numbers of organic producers [29].

With the exception of the nitrate research carried out by Brkić et al. [30] in 2017, this study was the first to include nitrite studies in the Split–Dalmatia County region. The present study was designed as a pilot effort to find out more regarding the nitrate and nitrite contents of vegetables grown locally.

## 2. Materials and Methods

### 2.1. Samples

**Figure 2:**
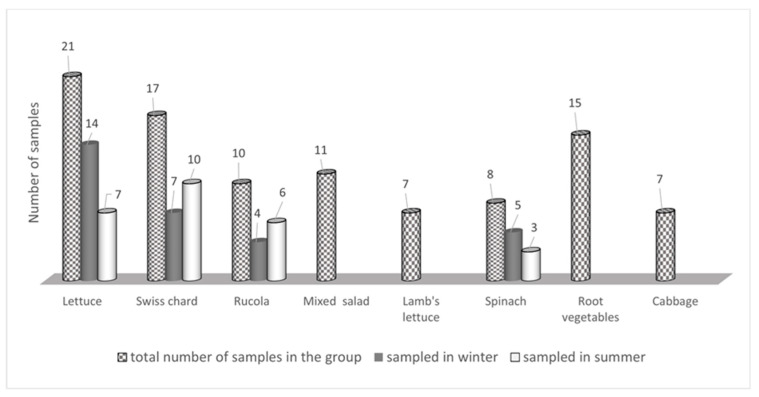
Distribution of the samples in the study, including the total number of samples within the group and the distribution by seasons.

In this study, we investigated a range of commonly eaten vegetables, some of which are not covered by nitrate and nitrite content regulations. In the period between June 2021 and January 2023, 96 samples of various vegetables were randomly collected from local stores in Split (Croatia) (Figure 2). Eight groups of samples were created due to the fact of their diversity and common characteristics: lettuce (*Lactuca sativa*); Swiss chard (*Beta vulgaris* L); rucola (*Eruca sativa* L.); mixed salad; Lamb’s lettuce (*Valerianella locusta*); spinach (*Spinacia oleracea*); root vegetables, including carrot (*Daucus carota*) and beetroot (*Beta vulgaris*); and cabbage (*Brassica oleracea*). Mixed salads, as the name implies, are unique meals composed of a variety of salads such as different varieties of lettuce or lettuce combined with chicory (*Cichorium intybus*). People frequently consume these mixtures in their raw, natural form, so for this reason, we included them in the study. Root vegetables (carrots and beets) are grouped together based on how they are prepared and consumed, because they are commonly consumed without any pretreatment (sinking or thermal treatment).

The sampling was carried out in two different seasons, winter and summer, to determine if there are differences in the nitrate and nitrite levels depending on the season. According to the collected data, the samples were cultivated on small farms in the vicinity of the sampling area. The cultivation conditions were not available. It is important to note that fresh vegetables were sampled (through sensory examination) to eliminate interference from deterioration caused by extended storing and rotting.

### 2.2. Chemicals and Working Standard Solutions

The octylamine, methanol, and standards sodium nitrate and sodium nitrite used in this study were purchased from Merck Co. (Darmstadt, Germany). Deionized water (0.05 μS cm^−1^) was prepared from the Ultrapure water system Omnia Pure (Stakpure GmbH, Germany). Standard stock solutions (1000 μg mL^−1^) were prepared by dissolving sodium nitrate and sodium nitrite in deionized water, while working solutions were prepared by serial dilution of the stock solution. The extraction solution was methanol:water = 1:1. The mobile phase was composed of methanol and 0.01 M octylamine (10:90, *v*/*v*, isocratic) [31]. Octylamine (0.01 M) was prepared by dissolving 1.29 g in 900 mL deionized water; the pH was adjusted to 7 with 10% phosphoric acid and filled up to 1 L.

### 2.3. Sample Preparation

Approximately 1 kg of each vegetable was sampled, taking care to select fresh vegetables without signs of wilting in order to avoid elevated results due to the fact of storage. The samples were prepared immediately upon receipt after removing parts that may have been damaged in transport. Before homogenization, the lower part of the leafy vegetable petiole was removed and the leaves were briefly washed under running water, while the root vegetables were peeled. In this way, possible traces of soil that could contribute to elevated nitrate results were removed. The sample preparation was carried out according to the procedure developed by Najdenkoska [31]. The entire mass of the sample was homogenized by a knife mill Grindomix GM 200 (Retsch, Germany). Five grams (±0.01) of homogenized sample were extracted with 100 mL MeOH:H_2_O = 1:1 extraction solution. The extraction was carried out in an ultrasonic bath for 15 min. The solution was filtered through a 0.45 µm syringe filter.

The majority of the nitrate and nitrite analyses were performed immediately after the sample preparation in order to eliminate possible interferences during the sample storage. Other samples were stored in the freezer (−20 °C) until the HPLC analyses, for a short period of no more than a few days.

### 2.4. Method

The quantitative and qualitative determination of the nitrates and nitrites in the vegetables was performed on an Agilent 1260 HPLC system (Agilent Technologies Singapore (International) Pte.Ltd., Singapore) equipped with a diode array detector (190–900 nm). The instrument was controlled via OpenLab CDS Chemstation Software (Agilent, rev.C.01.05(35)). For the separation, an Agilent Zorbax reversed-phase HPLC column (250 × 4.6 mm; 5 µm) was used with a flow of 1.1 mL/min and an injection volume of 10 µL. For both analytes, the maximum wavelength was 210 nm [31]. The limit of quantitation (LOQ) for both the nitrate and nitrate was 1.0 mg kg^−1^. The mean recovery was 96.0% for nitrate and 95.0% for nitrite, with a linearity (R^2^) of 0.999 for both analytes. All samples were analyzed in duplicate, with an RSD < 5%.

### 2.5. Statistical Analysis

The Kolmogorov–Smirnov test was used for the analysis of the distribution of normality. Since the distribution of our results did not follow a normal distribution, the Mann–Whitney U test and one-way ANOVA were used to investigate the differences among the levels in the two harvesting periods, whereby *p*-values less than 0.05 were considered statistically significant.

The nitrate and nitrite levels are expressed through arithmetic means, medians, and range. The arithmetic means of the nitrate and nitrite concentrations were used for comparison with the results of other studies. The SPSS v23 computer program (IBM Corp., Armonk, NY, USA) was used for descriptive and statistical data processing.

## 3. Results and Discussion

The findings of this study revealed elevated nitrate concentrations in vegetables that are unregulated by legislation. A large number of samples also contained nitrite, for which there is still no established maximum permitted level. The results of the nitrate and nitrite analyses in all samples, with included statistical variables, are summarized in Table 1 and Table 2.

In 90 samples (93.8%) the nitrate and in 27 samples (28.1%) the nitrite concentrations were above limit of quantitation (LOQ), that is, above 1 mg kg^−1^.

### 3.1. Nitrate Results

A review of the nitrate results (Table 1 and Figure 2) clearly shows that the highest results were obtained in the rucola samples, with no samples below the LOQ and 70% of the samples with concentrations that would exceed the ADI for nitrate if 100 g of vegetables were consumed daily.

We noticed that in 11 samples (five Swiss chard, five rucola, and one cabbage), based on a 100 g portion, the nitrate concentrations (ranged from 2659.4 to 4526.3 mg kg^−1^) exceeded the proposed ADI. In the samples of lettuce and spinach, there were also no samples below the LOQ, but the mean and median values were lower than they were in rucola. Assuming a serving size of 100 g, one sample from each group (one lettuce and one spinach) would exceed the ADI for nitrate.

This is not unusual since these three types of leafy vegetables are recognized as a significant vegetable source for nitrate. Regarding the limits set in Regulation 1258/2011 amending Regulation (EC) No. 1881/2006 as regards the maximum levels for nitrates in foodstuffs, all samples of lettuce, rucola, and spinach were consistent. Our results are within the results of similar studies [21,32,33,34,35,36].

A broad range of results can be observed within all sample groups, which probably is related to the type and frequency of the fertilizer application. According to data from the Faculty of Agriculture University of Zagreb, the region where the samples in this research were produced is characterized by a two times higher rate of use of artificial nitrogen fertilizers compared to organic nitrogen fertilizers [37].

**Figure 3:**
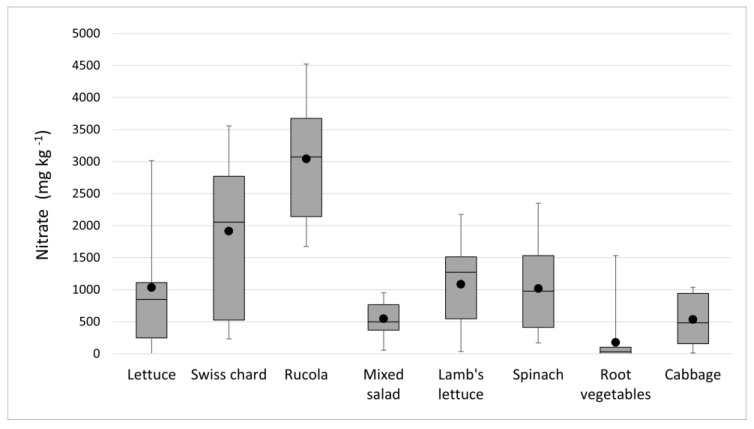
The results of the determination of the nitrate concentrations depending on the variables are shown as the mean value (•); maximum (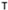); minimum (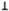); and interquartile range (with median (□).

The nitrate levels in the Swiss chard, for which there is no set maximum, are the findings that should unquestionably be discussed. The median and mean values of the nitrate concentrations found in the Swiss chard samples were two times higher in relation to the lettuce and spinach samples (Table 1). Furthermore, one sample of Swiss chard had nitrate amounts that exceeded the ones permissible in spinach; we compared those two types of vegetables for similarity in their preparation and consumption mode. The nitrate levels in Swiss chard are clearly greater than those in lettuce and spinach, as shown in Figure 3, with values above the median predominating. Swiss chard is very common in the diet in the Mediterranean region and represents a significant contribution to nitrate levels [38,39,40,41].

A common assumption that Swiss chard is consumed exclusively after cooking might bring up another possible issue in the exposure assessment. Namely, food processing (i.e., washing, peeling, blanching, and cooking) affects the nitrate levels in vegetables, with various effects. Nitrate is soluble in water, so the washing and soaking of leafy vegetables can reduce nitrate levels by 10–15%. Cooking and frying affect nitrate levels differently, usually 30–40%, but the results also depend on the type of vegetable [21,26,42,43]. Due to the fact of this, exposure evaluations frequently underestimate the nitrate contributions from Swiss chard by taking into consideration nitrate reduction factors related to the washing and cooking of vegetables.

On the other hand, in order to preserve different thermolabile vitamins and phytochemicals, the consumption of fresh food that has not been thermally processed is increasingly recommended [44]. Therefore, young, fresh Swiss chard is widely suggested by nutritionists and is a must for many proposed diets such as a vegetable salad or a smoothie. With such consumption, the effect of reducing nitrate levels due to the fact of heat treatment does not exist, leaving only a reduction for the washing process. This is another very important reason why agrotechnical measures should be followed during cultivation, and thereby result in lower nitrate levels in vegetables.

The acceptable daily intake is 0–3.7 mg of nitrate per kg b.w., corresponding to 222 mg for a person of 60 kg per day [21]. In our study, five samples of Swiss chard had nitrate concentrations in the range 2661.3–3558.4 mg kg^−1^. Even when reduction factors are taken into consideration (15% reduction for washing vegetables), a portion of 100 g of fresh Swish chard salad or smoothie had nitrate concentrations above the ADI of 222 mg.

Nitrate concentrations in rucola and lettuce samples in our research were lower, while the nitrate concentrations found in the Swiss chard and spinach samples were higher than those determined by the EFSA’s research [21]. One of the reasons could be that the frequency of Swiss chard consumption is underestimated in most of the exposure scenarios, since they are often based on the results of the official control. Swiss chard is not sampled at all in official controls, because no maximum levels are prescribed for it; therefore, assessments generally involve a limited number of samples gathered from other sources, such as local monitoring [21,41].

In addition, very few analytical results for nitrate concentrations in ready-to-eat vegetables are included in exposure assessments. Our results are similar to the results reported by Iammarino et al., although the maximum and mean nitrite concentrations in Swiss chard in his study were higher (5834.9 and 3063.2 mg kg^−1^), compared to 3558.4 and 1913.6 mg kg^−1^ in our study [33]. All these results indicate the necessity for amending the regulations and setting the maximum limits for Swiss chard.

**Figure 4:**
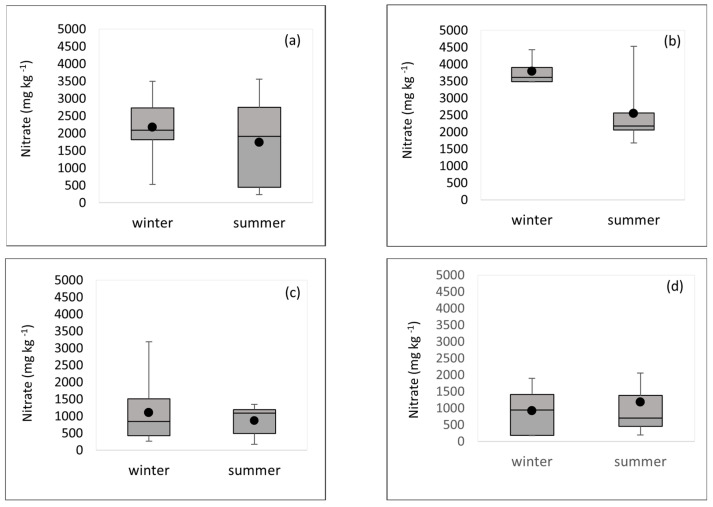
Distribution of the nitrate concentrations depending on the season: (**a**) Swiss chard; (**b**) rucola; (**c**) lettuce; (**d**) spinach.

We sampled vegetables during two different seasons, comparing the nitrate levels in samples cultivated through the winter and the summer season (mixed salads and Lamb’s lettuce and cabbage were only sampled in one season due to the fact of technical difficulties). Namely, the levels of nitrate in vegetables are affected by the intensity of light and, therefore, the concentrations differ depending on the season. Nitrate reductase activity decreases during the winter because of the reduced light intensity, leading to greater nitrate accumulation [21]. However, no statistically significant difference between summer and winter nitrate concentrations (*p* > 0.05) was found. The mean and median nitrate values in the Swiss chard, lettuce, and spinach samples harvested in winter and summer were very similar, which is opposite to rucola, where the nitrate levels were higher in the winter period (Figure 4).

This may be related to the geographical location of the area where the analyzed samples were cultivated, which had a mild Mediterranean climate and a large number of sunny days. Vegetables are mainly grown outside or in greenhouses with clear covers that allow enough light to pass through. The region is characterized with high-intensity irradiance throughout the year. Very similar results were found in the research of Brkić et al., who also analyzed samples from this area over two growing seasons [30]. Kyriacou et al. concluded in their study that the effect of irradiance in areas with many sunny days is significantly smaller compared to the influence of fertilization [45]. We did not have information on the cultivation data, type, and frequency of the fertilizers’ application. This is the limitation of our study, since we were not able to evaluate the impact of individual factors on the nitrate levels in the analyzed samples (type of fertilizer, frequency of fertilization, or environmental conditions, i.e., irradiance).

### 3.2. Nitrite Results

Most studies focus on the nitrate concentrations, as they are the main sources of nitrite. However, nitrite, which is potentially much more harmful, is not subject to any regulatory limitations in vegetables.

Our findings indicate that nitrite was present in 28.1% of the samples analyzed in our study, more precisely in 27 samples in the range of 2.7–537.9 mg kg^−1^. Twenty of those samples, were leafy vegetables intended for fresh consumption, (lettuce, mixed salads, corn salad, rucola, and spinach). In all samples of cabbage and root vegetables, the nitrite concentrations were below the LOQ. Within all other vegetable groups, the nitrite was found in various percentages of the samples. The samples of spinach and rucola had the highest mean values, while in the group of Swiss chard and mixed salad, the frequency of the samples with nitrite levels was the highest (Table 2).

**Figure 5:**
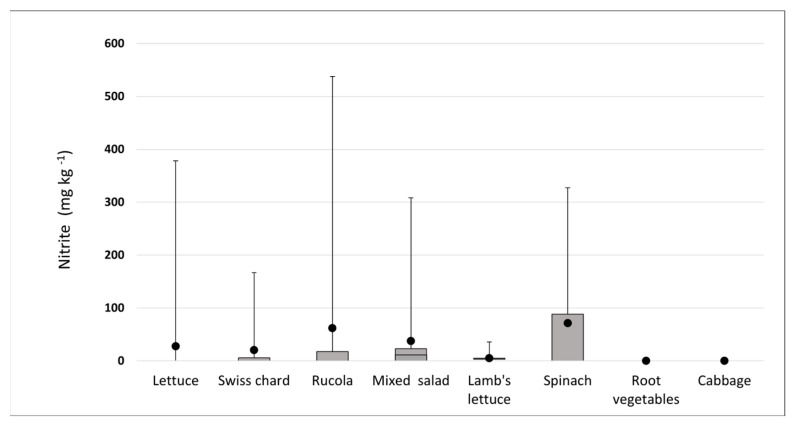
The results of the determination of the nitrite concentrations depending on the variables are shown as the mean value (•); maximum (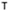); minimum (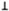); and interquartile range with median (□).

In several samples of rucola, lettuce, and spinach, the nitrite concentrations were very high: 537.9, 378.1, and 327.1 mg kg^−1^, respectively (Figure 5). These results need attention considering that there are no established legal limits for nitrite. Similar results were found in a study by Iammarino, who found elevated nitrite levels in spinach [32].

As noticed earlier, the samples of Swiss chard and mixed salad had the greatest prevalence of nitrite contamination with 41.2% and 54.5% above the LOQ. It must be emphasized that all samples in which nitrite was found also contained substantial levels of nitrate, which can be reduced after ingestion to produce endogenous nitrite. The results of the nitrite prevalence, as well as nitrite and nitrate results, are presented in Table 3.

According to JECFA, the acceptable daily intake is 0–0.07 mg of nitrite per kg b.w., corresponding to 4.2 mg for person of 60 kg per day [21]. Therefore, the ingestion of 100 g of raw rucola with a nitrite concentration of 540 mg kg−1 will result in an intake of 54 mg nitrite, which greatly exceeds the ADI. Assuming that the conversion factor of nitrate into nitrite is 7%, the potential risk of nitrites is even greater. The spinach samples in which high nitrite values were found (53.6, 192, and 327.1 mg kg^−1^) were spinach exclusively for fresh consumption.

Our findings indicate that leafy vegetables are a significant exogenous source of nitrite intake. Considering the recommendations for the increased consumption of fresh vegetables, such as smoothies and salads, these high concentrations highlight how important it is to control the concentration of nitrite in vegetables. Caution is especially necessary if such vegetables are consumed by children, and spinach is often present in their diet [46]. Namely, children have a higher stomach pH than adults and more nitrate-reducing bacteria in their intestinal flora. Due to the fact of this, they are exposed to a greater risk of the harmful effects of nitrite and the occurrence of methemoglobinemia [47]. Here again, it is necessary to emphasize the high levels (mean and median, Table 1) of nitrate in Swiss chard in our study. Swiss chard’s young leaves are also consumed fresh through salads and smoothies. Thus, all factors taken into account (reduction factor of 15% due to the preparation of vegetables and a conversion factor of 7% for the endogenous reduction of nitrate into nitrite), with 100 g of Swiss chard, a nitrite level exceeding the ADI is introduced into the body.

Considering the results of different studies, the question of the positive or negative effect of nitrates remains open [48]. Some results indicate a potentially beneficial effect of nitrates on lowering blood pressure and improving cognitive function [13,14,17,18]. Conversely, there are studies linking the consumption of nitrate-rich foods with a higher risk of type 2 diabetes, as well as gastrointestinal cancer [49,50]. Until this open question is resolved, it is important to be cautious and reduce the intake of harmful substances, especially for sensitive groups such as children. It is necessary to expand nitrate maximum levels for more types of vegetables, which are often represented in the diet. It is very important to set the maximum limits for nitrites as well, because they do not exist at all, although according to research results, their presence has been proven in vegetables. The question that arises is whether vegetables have to contain such high levels of nitrate and nitrite at all? A simple reduction in the use of artificial fertilizers would lead to a decrease in the levels of potentially harmful substances in vegetables, which would keep them a high-value food without the shadow of potential harm.

## 4. Conclusions

Nitrate and nitrite levels above the limit of quantification were found in a large amount of vegetables intended for fresh consumption. For some of them, such as Swiss Chard in which high nitrate concentrations were found, no legal limits have been established so far.

The samples with high nitrite levels, which significantly exceeded the recommended ADI values, are often present in children’s diets and in various diets based on fresh vegetables. Children are exposed to a greater risk of the possible harmful effects of nitrite and the occurrence of methemoglobinemia.

The results of our research support similar studies and indicate the importance of expanding existing maximum limits and to cover more types of vegetables. It is also important to set maximum limits and establish control of nitrite levels in vegetables.

Our study has a limitation due to the lack of cultivation data; therefore, we could not assess with certainty how much seasonality affects nitrate levels in vegetables, although we did not find a statistically significant difference between summer and winter harvests.

Although various studies are being conducted on the possible beneficial effects of nitrates and nitrites, there are still many controversies among the results.

This research is planned to be continued and expanded with data on the impact of different preparation procedures on nitrite levels, with an emphasis on vegetables intended for fresh consumption.

## Figures and Tables

**Table 1:** Distribution and levels of nitrate (mg kg^−1^) in the different types of vegetables in this research.

Sample	No. of Samples	Mean ± SD (mg kg^−1^)	Median (mg kg^−1^)	Concentration Range (mg kg^−1^)	Maximum Levels (mg kg^−1^) (Regulation 1258/2011)	
					Harvested 1.10.–30.3.	Harvested 1.04.–30.9.
Lettuce	21	1035.3 ± 732.6	1020.1	169.7–3184.2	5000 ^(UC)^ 4000 ^(OA)^	4000 ^(UC)^ 3000 ^(OA)^
Swiss chard	17	1913.6 ± 1126.6	2055.4	233.4–3558.4	-	
Rucola	10	3041.4 ± 974.7	3073.2	1674.8–4526.3	7000	6000
Mixed salad	11	548.0 ± 269.1	199.2	54.6–952.6	-	
Lamb’s lettuce	7	1086.5 ± 703.5	1273.8	36.1–2175.5	-	
Spinach	8	1056.5 ± 742.0	976.8	168.5–2350.9	3500	
Root vegetables	15	178.8 ± 396.4	32.2	<LOQ–1534.8	-	
Cabbage	7	533.6 ± 406.0	484.1	13.5–1040.1	-	
Total	96					

SD, standard deviation; UC, grown under cover; OA, grown in the open air; LOQ, limit of quantitation = 1 mg kg^−1.^

**Table 2:** Distribution and levels of nitrite (mg kg^−1^) in the different types of vegetables in this research.

Sample	Number of Samples	Mean ± SD (mg kg^−1^)	Median (mg kg^−1^)	Concentration Range (mg kg^−1^)	Concentration Range in Samples > LOQ (mg kg^−1^)
Lettuce	21	27.7 ± 81.9	0.0	<LOQ—378.1	21.1–378.1
Swiss chard	17	20.7 ± 43.0	0.0	<LOQ—167.0	4.1–167.0
Rucola	10	61.9 ± 159.6	0.0	<LOQ—537.9	2.7–537.9
Mixed salad	11	37.5 ± 86.5	11.3	<LOQ—308.6	11.3–308.6
Lamb’s lettuce	7	5.1 ± 11.2	0.0	<LOQ—32.5	3.3–32.5
Spinach	8	71.6 ± 115.0	0.0	<LOQ—327.1	53.6–327.1
Root vegetables	15	0.0	0.0	<LOQ	0
Cabbage	7	0.0	0.0	<LOQ	0
Total	96				

LOQ, limit of quantitation = 1 mg kg^−1.^

**Table 3:** Prevalence of the nitrite concentrations with accompanying nitrate concentrations in these samples.

No. of Samples with NO_2_ above the LOQ		%		Samples with Nitrite Concentrations above the LOQ (1 mg kg^−1^)						
			mg kg^−1^	1	2	3	4	5	6	7
Lettuce	5	23.8	nitrate nitrite	764.0	262	747.7	1157.6	3184.2		
				98.9	55.9	378.1	28.4	21.1		
Swiss chard	7	41.2	nitrate	2408.3	3055.9	242.4	233.4	2661.3	3558.4	1571.0
			nitrite	30.4	57.6	167.0	6.1	80.4	5.8	4.1
Rucola	4	40.0	nitrate	4427.6	2046.6	2262.4	4526.3			
			nitrite	22.9	55.2	537.9	2.7			
Mixed salads	6	54.5	nitrate	653.8	952.6	54.6	577.9	908.2	882.3	
			nitrite	31.6	14.7	308.6	11.3	16.6	29.3	
Lamb’s lettuce	2	28.6	nitrate	1647.6	1382.9					
			nitrite	3.3	32.5					
Spinach	3	37.5	nitrate	2350.9	489.8	1006.2				
			nitrite	192.0	327.1	53.6				

LOQ, limit of quantitation = 1 mg kg^−1^.

## Data Availability

The data presented in this study are available upon request from the corresponding author.
